# Plasma intact fibroblast growth factor 23 levels in women with anorexia nervosa

**DOI:** 10.1186/1751-0759-2-10

**Published:** 2008-04-16

**Authors:** Makoto Otani, Yoshiyuki Takimoto, Junko Moriya, Kazuhiro Yoshiuchi, Akira Akabayashi

**Affiliations:** 1 Department of Stress Sciences and Psychosomatic Medicine, The University of Tokyo, 7-3-1 Hongo, Bunkyo-ku, Tokyo 113-8655, Japan

## Abstract

**Background:**

Fibroblast growth factor (FGF)23 is a novel phosphaturic factor associated with inorganic phosphate homeostasis. Previous human studies have shown that serum FGF23 levels increase in response to a high phosphate diet. For anorexia nervosa (AN) patients, inorganic phosphate homeostasis is important in the clinical course, such as in refeeding syndrome. The purpose of this study was to determine plasma levels of intact FGF23 (iFGF23) in restricting-type AN (AN-R) patients, binge-eating/purging-type AN (AN-BP) patients, and healthy controls.

**Methods:**

The subjects consisted of 6 female AN-R patients, 6 female AN-BP patients, and 11 healthy female controls; both inpatients and outpatients were included. Plasma iFGF23, 1,25-dihydroxyvitamin D (1,25-(OH)_2_D), and 25-hydroxyvitamin D (25-OHD) levels were measured. Data are presented as the median and the range. A two-tailed Mann-Whitney U-test with Bonferroni correction was used to assess differences among the three groups, and a value of p < 0.017 was considered statistically significant.

**Results:**

There were no differences between AN-R patients and controls in the iFGF23 and 1,25-(OH)_2_D levels. In AN-BP patients, the iFGF23 level (41.3 pg/ml; range, 6.1–155.5 pg/ml) was significantly higher than in controls (3.8 pg/ml; range, not detected-21.3 pg/ml; p = 0.001), and the 1,25-(OH)_2_D was significantly lower in AN-BP patients (7.0 pg/ml; range, 4.2–33.7 pg/ml) than in controls (39.7 pg/ml; range, 6.3–58.5 pg/ml; p = 0.015). No differences in plasma 25-OHD levels were observed among the groups.

**Conclusion:**

This preliminary study is the first to show that plasma iFGF23 levels are increased in AN-BP patients, and that these elevated plasma FGF23 levels might be related to the decrease in plasma 1,25-(OH)_2_D levels.

## Findings

Fibroblast growth factor (FGF)23, a circulating 26 kDa peptide produced by osteogenic cells, is a novel phosphaturic factor. It is important for the regulation of inorganic phosphate homeostasis and for vitamin D metabolism [1]. FGF23 inhibits renal proximal tubule phosphate reabsorption, increases renal phosphate excretion, and reduces serum phosphate without affecting serum calcium. FGF23 also strongly suppresses 1,25-(OH)_2_D production [2,3].

Anorexia nervosa (AN) is an eating disorder characterized by decreased caloric intake, low weight, and reduced body fat. To date, two subtypes have been identified: restricting-type (AN-R); and binge-eating/purging-type (AN-BP). AN is diagnosed by weight loss and refusal to maintain a minimal normal body weight, an intense fear of gaining weight or becoming fat, a self-evaluation unduly influenced by body shape and weight, and amenorrhea [4]. AN-R patients restrict food intake, while AN-BP patients regularly engage in binge-eating and/or purging.

In patients with AN, refeeding syndrome is a well-known phenomenon that occurs during the course of nutritional rehabilitation; it is characterized by hypophosphatemia, which may result in serious consequences, such as cardiac dysrhythmia, delirium, and even sudden death [5]. Although inorganic phosphate homeostasis is important in AN patients, no previous studies have examined plasma FGF23 levels in AN. Therefore, the present study determined plasma FGF23 concentrations in AN-R patients, AN-BP patients, and healthy controls.

The subjects included 12 female AN patients who met the diagnostic criteria of the Diagnostic and Statistical Manual of Mental Disorders-Fourth Edition (DSM-IV) [4] and 11 healthy female controls. The 12 AN patients included 6 patients with AN-R and 6 patients with AN-BP. No patients had a previous diagnosis of bulimia nervosa. The study's cases included outpatients and inpatients of the University of Tokyo Hospital. Except for proper doses of antidepressants, anxiolytics, hypnotics, laxatives and stomach agents, patients with AN-BP were treated with lactomin (3 g/day; n = 1), lomerizine (10 mg/day; n = 1) and pantethine (300 mg/day; n = 1), and AN-R patients and controls did not receive drug therapy. Premorbid renal dysfunction was an exclusionary criterion.

Blood samples were collected from all subjects after overnight fasting. The protocol was approved by the Institutional Ethics Committee of the University of Tokyo, and written informed consent was obtained from all subjects prior to enrollment in the study.

All blood samples were drawn into chilled tubes containing EDTA-2Na (1 mg/ml) and were then immediately centrifuged at 4°C. Plasma portions were stored at -70°C prior to analysis. Plasma concentrations of intact FGF23 (iFGF23) were measured using an ELISA kit (Immutopics, San Clemente, CA, USA) (6,7), with a sensitivity of 1.0 pg/ml, intra-assay variability of <4.4%, and inter-assay variability of <6.5%. All samples were analyzed in duplicate. Plasma 1,25-(OH)_2_D and 25-OHD concentrations were measured using RIA (SRL, Tokyo, Japan). Plasma calcium and phosphate concentrations were measured using standard laboratory methods (SRL).

A two-tailed Mann-Whitney U-test with Bonferroni correction was done after Kruskal-Wallis testing to assess differences among the three groups. A usual two-tailed Mann-Whitney U-test was used to assess differences between AN-R patients and AN-BP patients when healthy controls were missing data points. Values of p < 0.05 were considered statistically significant on the Kruskal-Wallis test and on the usual two-tailed Mann-Whitney U-test, and values of p < 0.017 were considered statistically significant on the two-tailed Mann-Whitney U-test with Bonferroni correction. Spearman's rank-correlation coefficients (ρ) was used to assess the relationship between iFGF23 and age and body mass index (BMI) for AN patients. All statistical calculations were performed using SPSS for Windows version 10.0 (SPSS, Chicago, IL, USA). All data are presented as the median and range.

Clinical profiles and biochemical data are summarized in Table 1.

**Table 1:** Clinical profiles and biochemical data of women with anorexia nervosa and healthy controls

	AN-R (n = 6)	AN-BP (n = 6)	Controls (n = 11)	P
Body Mass Index (kg/m^2^)	16.0 * (13.8–17.4)	13.8 * (13.0–15.5)	19.7 (18.5–23.7)	<0.001
Age (years)	19 (17–32)	31 (19–38)	27 (21–32)	0.034
age at the time of disease onset (years)	17 (16–30)	24 (14–34)		
disease duration (yaers)	1.8 (0.5–3.0)	5.0 § (2.0–11.0)		
frequency of binge eating	None (none-none)	3.5/week (none-2–3/day)		
frequency of vomiting	None (none-none)	1/week § (none-2–3/day)		
Ca (mg/dl)	10.2 * ‡ (9.5–10.7)	9.1 (7.9–9.4)	8.9 (8.4–9.6)	0.018
P (mg/dl)	3.4 (3.1–4.2)	3.5 (3.0–4.0)	2.9 (2.0–3.7)	0.029
1,25-(OH)_2_D (pg/ml)	34.4 (19.0–51.5)	7.0 * † (4.2–33.7)	39.7 (6.3–58.5)	0.023
25-OHD (ng/ml)	26 (19–30)	18 (11–31)	20 (12–28)	NS

All values are described as median (minimum-maximum).

P values in the rightmost column were calculated using Kruskal-Wallis tests.

* p < 0.017 vs. controls, using a two-tailed Mann-Whitney U-test with Bonferroni correction.

† p < 0.017 vs. AN-R, using a two-tailed Mann-Whitney U-test with Bonferroni correction.

‡ p < 0.017 vs. AN-BP, using a two-tailed Mann-Whitney U-test with Bonferroni correction.

§ p < 0.05 vs. AN-R, using a usual two-tailed Mann-Whitney U-test.

Plasma calcium levels in AN-R patients were significantly increased when compared with controls (p < 0.001), while there were no differences between AN-BP patients and controls (p = 0.350). No statistically significant differences in plasma phosphate levels were observed among the groups.

**Figure 1:**
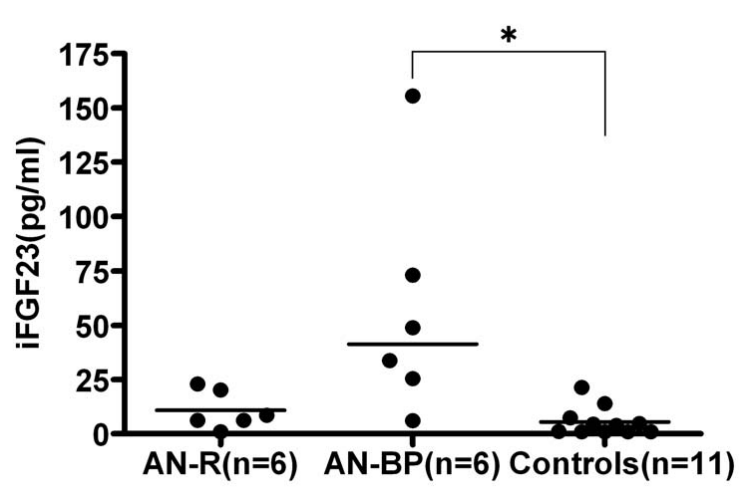
**Dot plots of plasma iFGF23 levels in AN patients and healthy controls.** The graphs depict median values (bars). Two-tailed Mann-Whitney U-tests with Bonferroni correction were used to assess differences among groups. A value of p < 0.017 was considered statistically significant. *p < 0.017 vs. controls.

Plasma iFGF23 levels were significantly greater in AN-BP patients than in controls (p = 0.001); there was no difference between AN-R patients and controls (p = 0.149; fig. 1). Plasma iFGF23 levels tended to be higher in AN-BP than in AN-R patients, but this difference was not statistically significant (p = 0.041). For AN patients, iFGF23 values did not correlate significantly with age (ρ = 0.181, p = 0.574) or with BMI (ρ = -0.112, p = 0.728).

No differences in plasma 25-OHD levels were observed among the groups. Plasma 1,25-(OH)_2_D was significantly lower in AN-BP patients than in controls (p = 0.015) and in AN-R patients (p = 0.015); there was no difference between AN-R patients and controls (p = 0.733).

This is the first study to show that AN-BP patients have elevated plasma iFGF23 levels. Plasma iFGF23 levels were significantly greater in AN-BP patients than in healthy controls, while there was no difference in plasma iFGF23 levels between AN-R patients and controls.

Previous reports showed that phosphate restriction significantly reduced FGF23 concentrations in healthy men and women [8-10]. In patients with severe malnutrition and a very limited phosphate intake, phosphate restriction is considered to be responsible for decreased FGF23 levels. In this study, the AN-R patients were all being treated as inpatients or outpatients; therefore, they might have had a certain amount of phosphate intake. This fact might have contributed to the lack of a difference in iFGF23 levels between AN-R patients and controls in the present study.

Interestingly, plasma iFGF23 levels were significantly higher in AN-BP than in healthy controls, while there were no differences in plasma phosphate levels among the groups. During binge eating, AN-BP patients eat a large quantity of food at once, including foods such as chocolates, cakes, snacks, and sweet buns, which generally contain moderate to large amounts of phosphate. In other words, binge eating in AN-BP patients might be regarded as acute phosphate loading. Previous reports found that, in healthy men and women, FGF23 concentrations were significantly increased by phosphate loading [9,10]; in healthy men, serum iFGF23 increased significantly 8 h after intake of 1,200 mg phosphate, compared to 8 h after intake of 400 mg and 800 mg phosphate [11]. Thus, our findings support the idea that AN-BP patients, most of whom have regularly engaged in binge eating, have increased plasma FGF23 levels due to the acute phosphate loading that occurs with binge eating. No previous reports have described the effects of binge eating on plasma FGF23 levels.

An earlier report showed that frequent vomiting increased serum amylase levels in AN patients [12]. The serum amylase level is an established indicator of vomiting behavior in AN patients. However, currently there is no established indicator for binge eating behavior. This preliminary study implies that plasma FGF23 levels might be a suitable candidate as an indicator of binge eating in AN patients.

The present study also found that AN-BP patients had a significantly lower plasma 1,25-(OH)_2_D level than both healthy controls and AN-R patients. Injection of recombinant FGF23 into normal and parathyroidectomized animals caused a reduction in serum 1,25-(OH)_2_D levels [2]. Elevated plasma FGF23 in AN-BP patients might decrease plasma 1,25-(OH)_2_D levels.

Conflicting results have been reported regarding 1,25-(OH)_2_D levels in AN patients. Some reports have shown significantly lower serum or plasma 1,25-(OH)_2_D levels in AN patients [13,14], while others have reported that AN patients have normal 1,25-(OH)_2_D levels [15]. However, in these previous reports, the subjects were not categorized into AN-R and AN-BP groups. In the present study, plasma 1,25-(OH)_2_D levels in AN-BP patients were significantly lower than in AN-R patients. Our results indicate the need for investigations that differentiate between AN subtypes.

The present study has three limitations. First, the number of AN patients was extremely small. Second, the volume of binge eating and purging in the AN-BP patients before participation in the study was not available. We were therefore unable to completely assess whether binge eating primarily affects plasma FGF23 levels in AN patients. Third, inorganic phosphate intake prior to participation in the study was not assessed in AN-R patients. In future studies, in addition to plasma FGF23, 1,25-(OH)_2_D and 25-OHD levels, inorganic phosphate intake, and the volume of binge eating prior to participation in the study should be determined.

This preliminary study showed for the first time that plasma iFGF23 levels are increased in AN-BP patients, and that these elevated plasma FGF23 levels might decrease plasma 1,25-(OH)_2_D levels. In AN-BP patients, plasma FGF23 levels might be an indicator of binge-eating behavior, which is characterized by acute phosphate intake.

## Competing interests

The author(s) declare that they have no competing interests.

## Authors' contributions

MO designed the study, analyzed the data, performed the statistical analysis, interpreted the results, and drafted the manuscript. JM collected the data. YT, KY and AA helped analyze the data, interpret the results, and draft the manuscript. All authors read and approved the final manuscript.
